# Supplementary material for: Faster Cryptococcus Melanization Increases Virulence in Experimental and Human Cryptococcosis
Source: J Fungi (Basel). 2022 Apr 12;8(4):393. doi: 10.3390/jof8040393 (PMC9029458; doi:10.3390/jof8040393)
Supplement: Supplementary file 1 [file jof-08-00393-s001.zip › Supplementary figures.pdf]

Supplementary figures

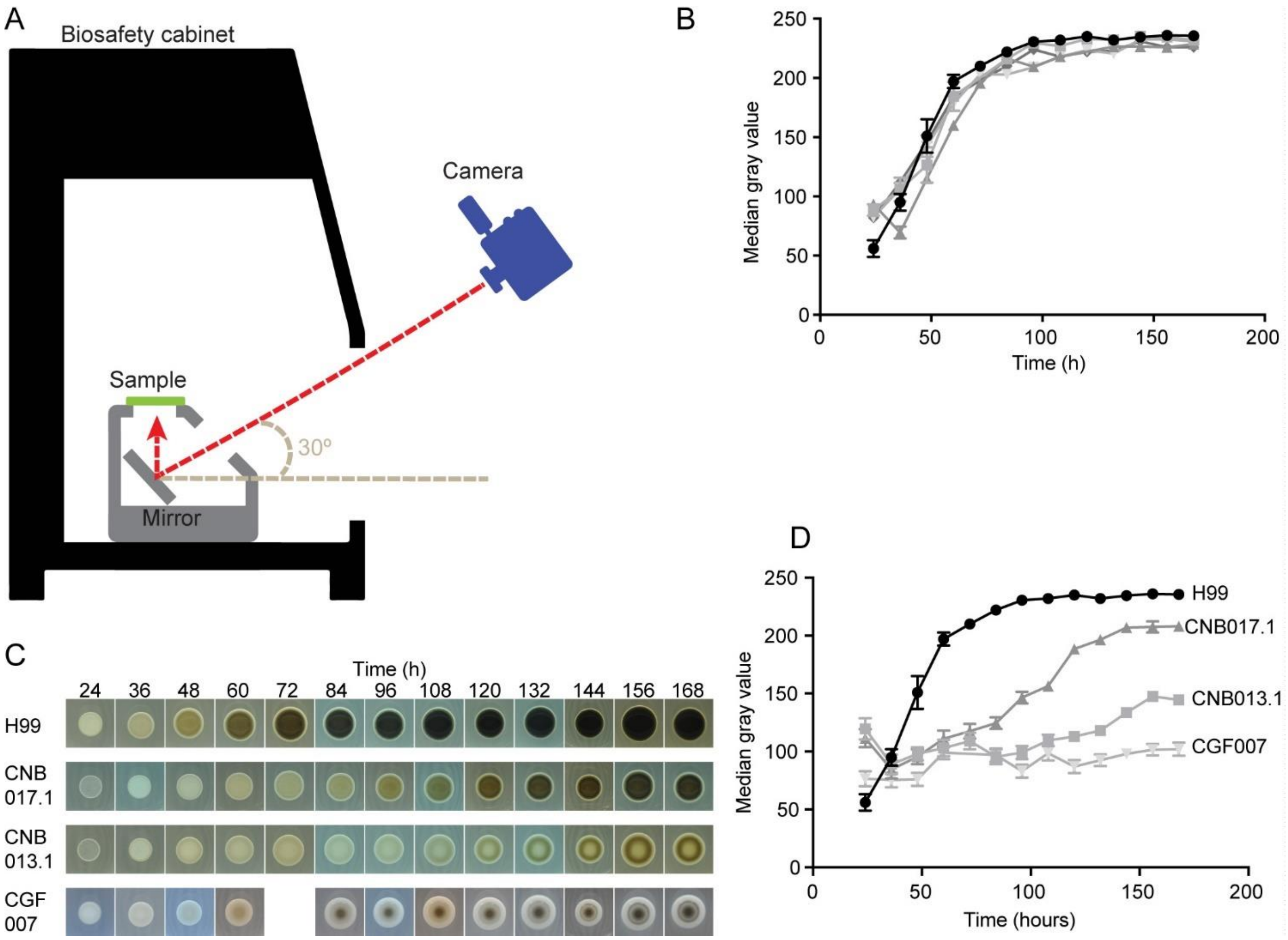

Figure S1. Quantifying colony melanization kinetics. (**A**) Colony image capture system using a mirror plate reflector device developed and manufactured in our research group. (**B**) Five biological replicates of H99 performed in duplicate demonstrating this method's high reproducibility. Three image processing and statistical analysis programs (Adobe Photoshop CC version 19.0, ImageJ version 1.50i and Prism 7 version 7.0a, respectively) were used to calculate the median gray values of each colony over time. (**C**) Representative photos of the macroscopic melanization profile of three clinical isolates and the standard H99 internal control strain in all experiments. Two strains with homogeneous colony melanization (H99 and CNB017.1) and two with a heterogeneous pattern (CNB013.1 and CGF007) are shown. (**D**) Non-linear regression curves of median gray values of the three isolates depicted on panel **C**.

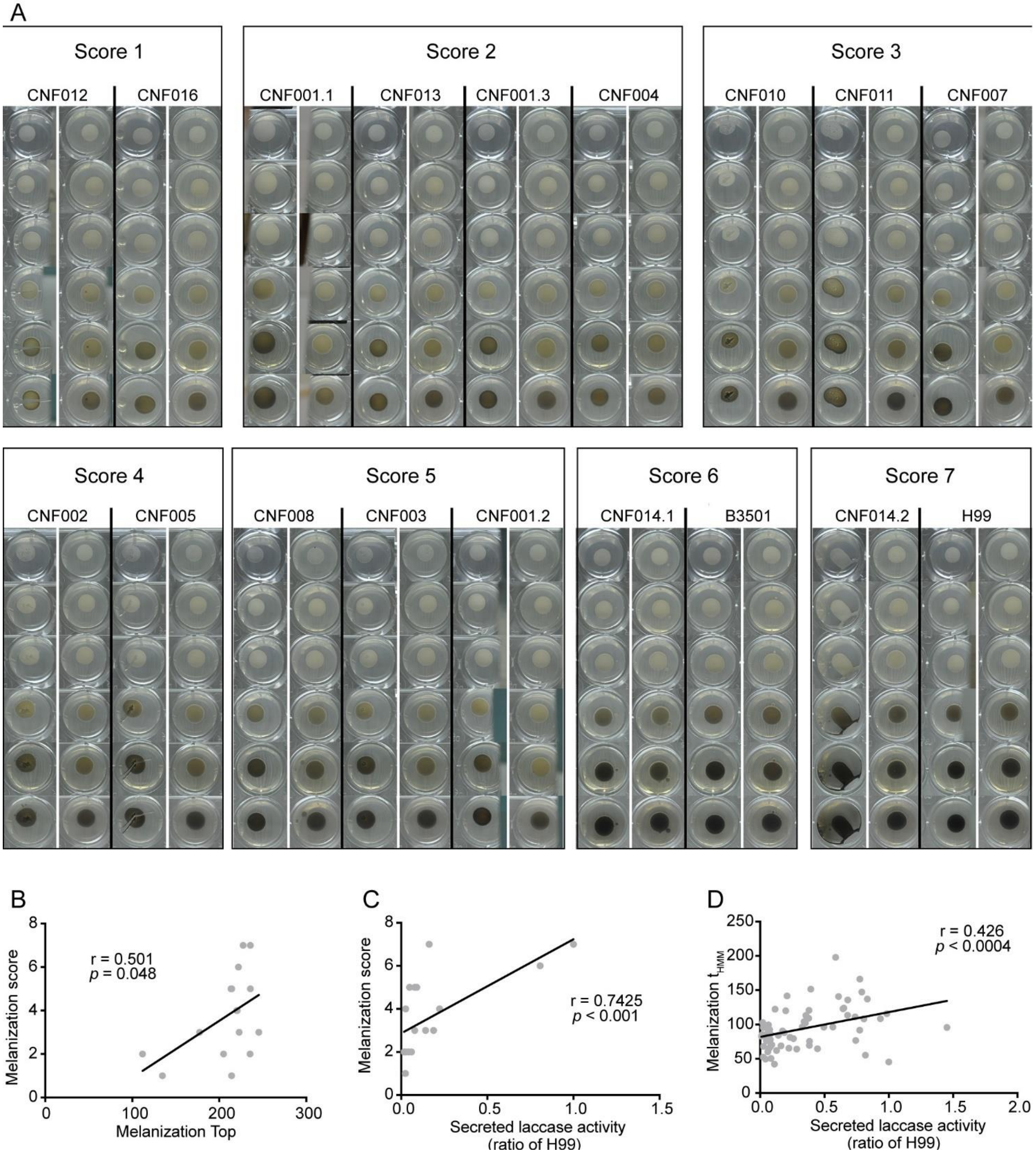

Figure S2. Validation of the densitometric method to measure melanization. (**A**) Semi-quantitative melanization score. The isolates were qualitatively categorized into 7 groups ordered from 1 to 7. Group 1 contains the isolates whose colonies had the least pigment and melanized slowest and group 7 those isolates that had the fastest and most intense melanin production. (**B**) Correlation between Melanization Top (maximum melanization index from non-linear regression curve of median gray value) and the semi-quantitative melanization score. (**C**) Correlation between secreted laccase activity and the semi-quantitative melanization score. (**D**) Correlation between secreted laccase activity and the time to half-maximum melanization t_{HMM}. The semi-quantitative melanization score was defined in parallel by two experienced individuals who were blinded to the other's judgment. All correlations were made with Spearman rank.

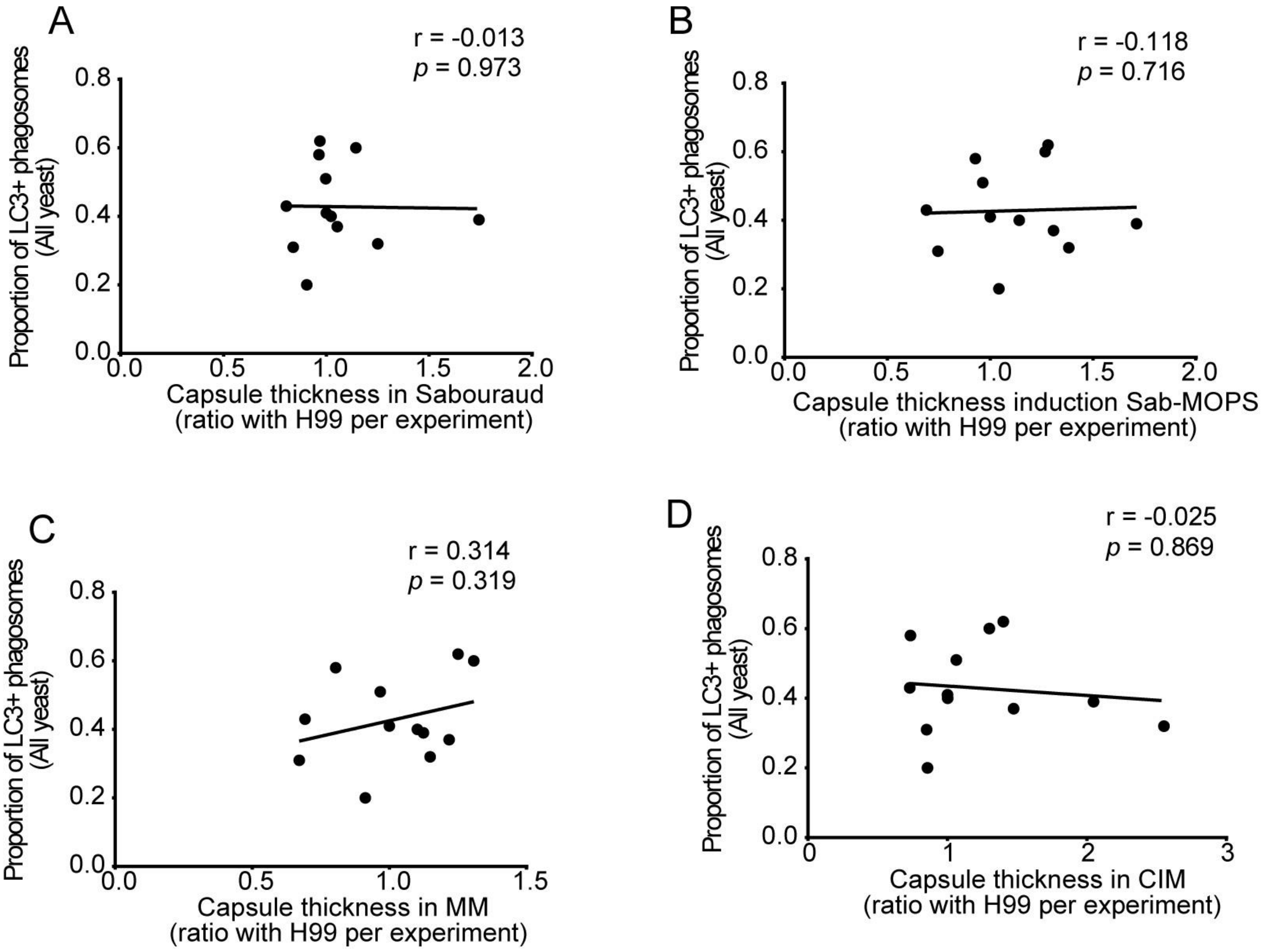

Figure S3. Capsule thickness does not affect the ability of clinical isolates to escape from LC3-associated phagocytosis. No correlation was found between LC3-associated phagocytosis (defined as the percentage of macrophages in which all internalized yeasts were in LC3-positive vacuoles among all macrophages with at least one phagocytosed yeast cell) and capsule thickness in the rich Sabouraud medium (**A**) and the capsule induction media Sab-MOPS (**B**), minimal medium (**C**) and CO_2 independent medium (**D**). All correlations were made with Spearman rank.
